# Primary Tuberculosis of the Cuboid and Fifth Metatarsal Without Pulmonary Involvement: A Rare Case Report

**DOI:** 10.7759/cureus.43049

**Published:** 2023-08-06

**Authors:** Sankalp Yadav, Gautam Rawal, Madhan Jeyaraman

**Affiliations:** 1 Medicine, Shri Madan Lal Khurana Chest Clinic, New Delhi, IND; 2 Respiratory Medical Critical Care, Max Super Speciality Hospital, New Delhi, IND; 3 Orthopaedics, ACS Medical College and Hospital, Dr. MGR Educational and Research Institute, Chennai, IND

**Keywords:** cbnaat/ xpert/ rif assay, tuberculosis, fifth metatarsal, cuboid, mtb (mycobacterium tuberculosis)

## Abstract

Tuberculosis affecting the smaller bones of the foot is relatively infrequent. There is a paucity of data related to the tubercular involvement of cuboid and metatarsal bones. Diagnosis is challenging, especially in the absence of pulmonary involvement, a history of trauma, and contact with tuberculosis. We present a rare case of primary tuberculosis of the cuboid and fifth metatarsal without pulmonary involvement. The patient was started on anti-tubercular chemotherapy. A detailed literature search revealed that no such case with concomitant involvement of the cuboid and fifth metatarsal without pulmonary seeding has ever been reported.

## Introduction

Tuberculosis is a global public health scare that mainly affects the countries of Asia, Africa, and Europe [1,2]. Tuberculosis is a result of infection by *Mycobacterium tuberculosis*, or Koch's bacillus, and belongs to the family Mycobacteriaceae [3]. Usually, the deposition of bacteria in the lung alveoli due to the inhalation of infected aerosols results in the disease [1]. However, this is not always true, as direct seeding through the broken skin could result in a local infection [4].

Extrapulmonary tuberculosis is comparatively rare, accounting for only 10-15% of all tuberculosis cases [5]. Further, tubercular involvement of the musculoskeletal system is reported in only 1-5% of patients with tuberculosis, which is nearly 10-18% of all cases of extrapulmonary tuberculosis [6,7]. Tubercular involvement of the small bones of the foot is rarely reported, even in endemic settings [8]. It is mainly reported in the talus, distal end of the first metatarsal, navicular, cuneiform, and cuboid bones [1,9].

Herein, a 34-year-old Indian male presented with pain and swelling in his right foot. He underwent a battery of lab investigations along with radiographs to establish the diagnosis. Finally, he was managed with anti-tubercular treatment.

## Case presentation

A 34-year-old, non-diabetic Indian male reported complaints of pain and swelling in his right foot for two months. He was asymptomatic two months ago when he had pain in the midfoot. This was followed by swelling on the dorsum of the foot. The pain was initially mild but increased to impact his walk, resulting in a visible limp. His pain subsided for a short while after taking an over-the-counter nonsteroidal anti-inflammatory drug. There was no fever, weight loss, cough, or night sweats. He was a daily wager with no history of smoking, alcoholism, or any substance abuse. Further, he and his contacts did not have a background of tuberculosis or any other medical or surgical illness. In addition, there was no history of trauma or staying at refugee camps, prisons, or night shelters.

**Figure 1:**
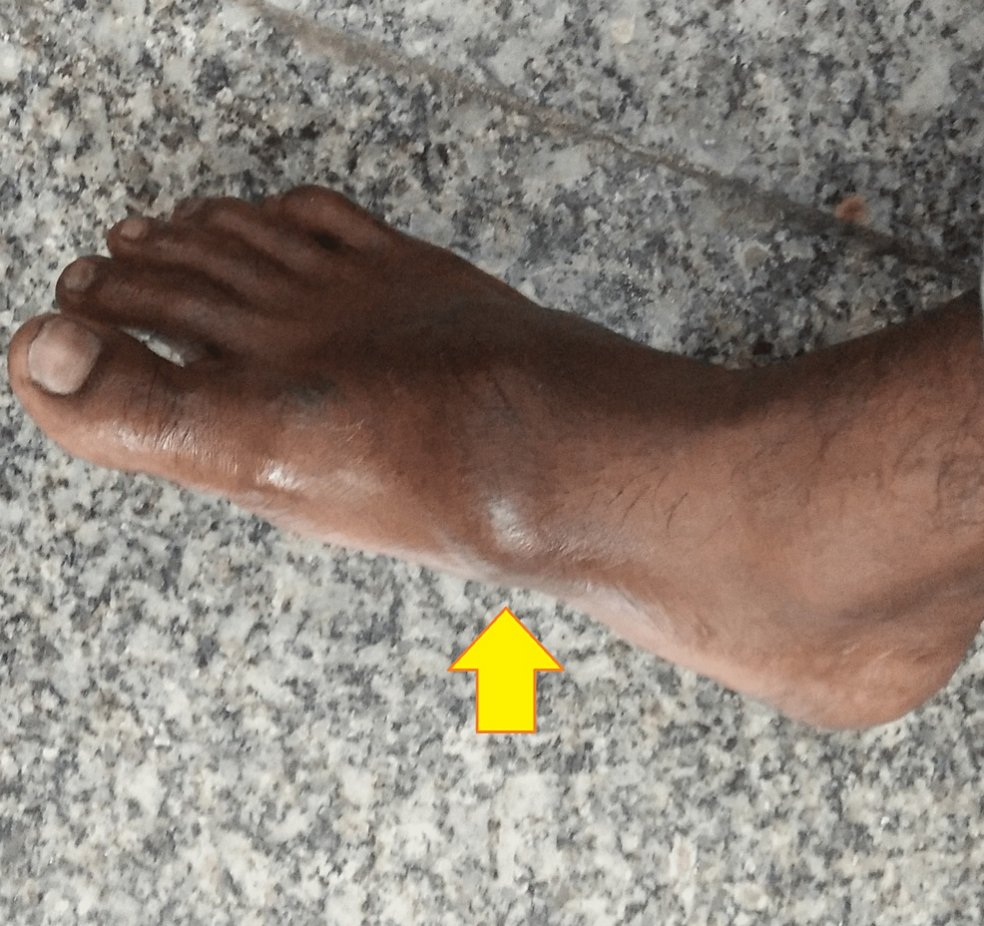
Gross image of the right foot showing swelling over the entire dorsum

A general examination indicated a hemodynamically stable man with a medium build. His systemic examination was unremarkable. A local examination of the right foot was remarkable for a 6x6 cm swelling over the entire dorsum of the right foot with a relatively smooth surface and tenderness on deep pressure over the fifth metatarsal bone (Figure 1).

The adjoining skin had no dilated veins, ulcers, or discharging sinuses. Moreover, the swelling was localized over the midfoot and was not associated with aggravating or relieving factors. There were limited eversion and inversion movements of the right foot, with painful dorsiflexion and plantar flexion terminally. His left foot was normal. Nevertheless, there was no clubbing, cyanosis, icterus, pallor, koilonychia, or lymphadenopathy.

**Figure 2:**
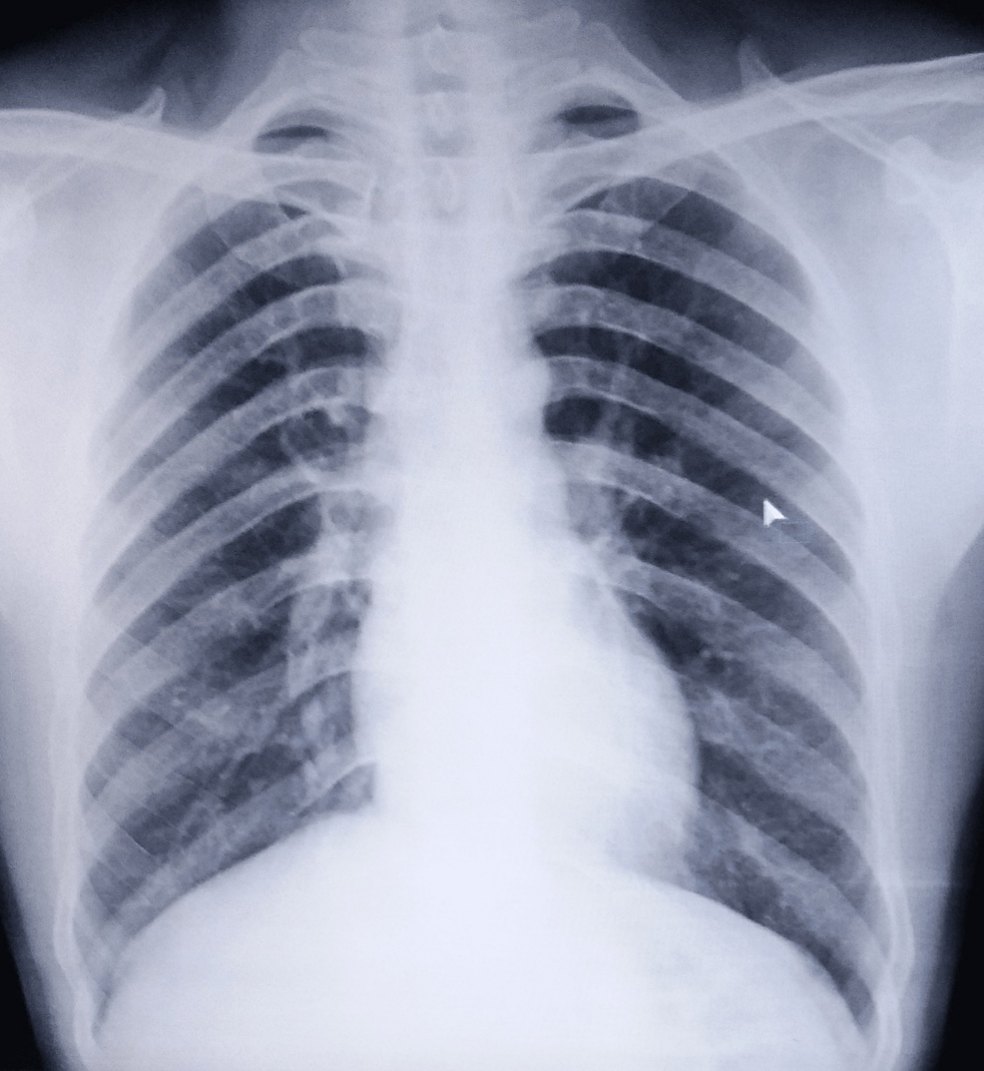
A chest radiograph not suggestive of pulmonary tuberculosis

A tentative diagnosis of pyogenic osteomyelitis was made with differentials for tuberculosis, bone tumor, fungal osteomyelitis, and granulomatous diseases such as gout. He was also advised routine blood investigations, induced sputum for an acid-fast bacilli test, a chest radiograph, and a radiograph of the right foot. Serological markers, sputum smear microscopy, and cartridge-based nucleic acid amplification tests of induced sputum were unremarkable. His HIV (I and II) were non-reactive. Additionally, there were no abnormalities on his chest radiograph (Figure 2).

**Figure 3:**
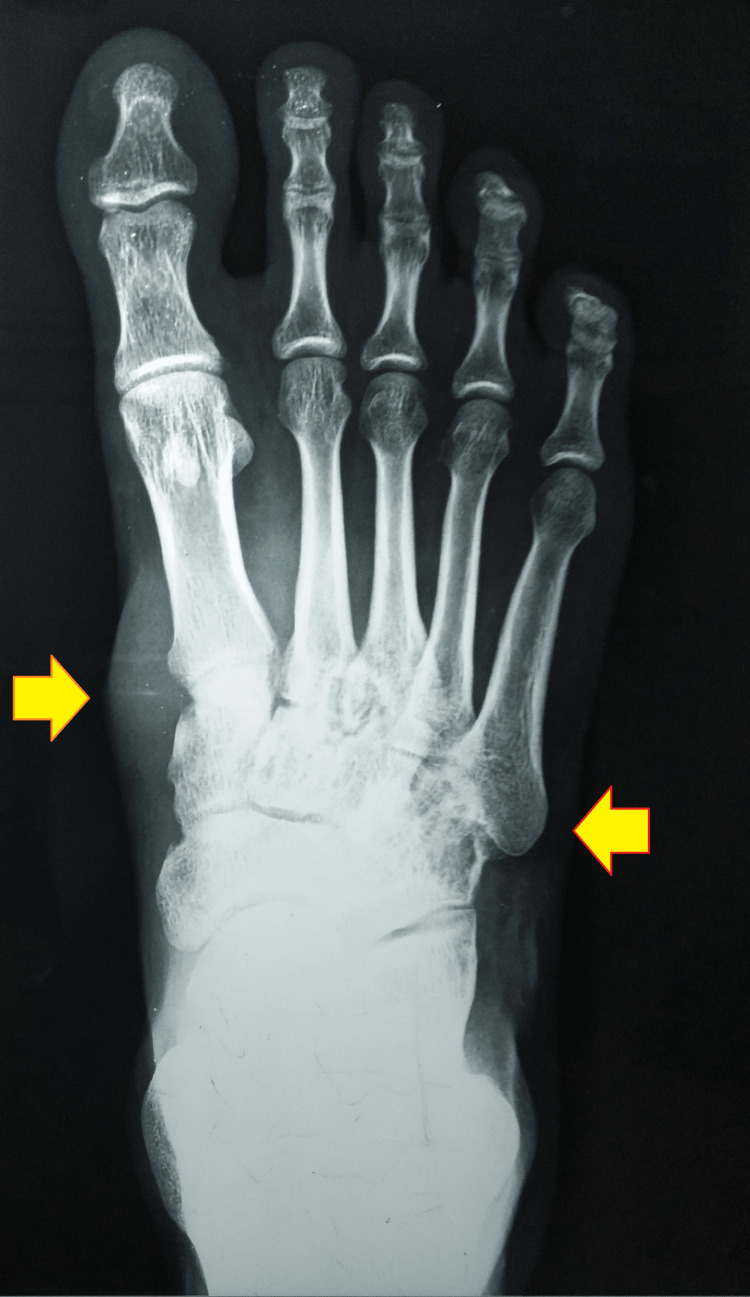
Anteroposterior radiograph of the right foot showing soft tissue swelling and bony involvement

**Figure 4:**
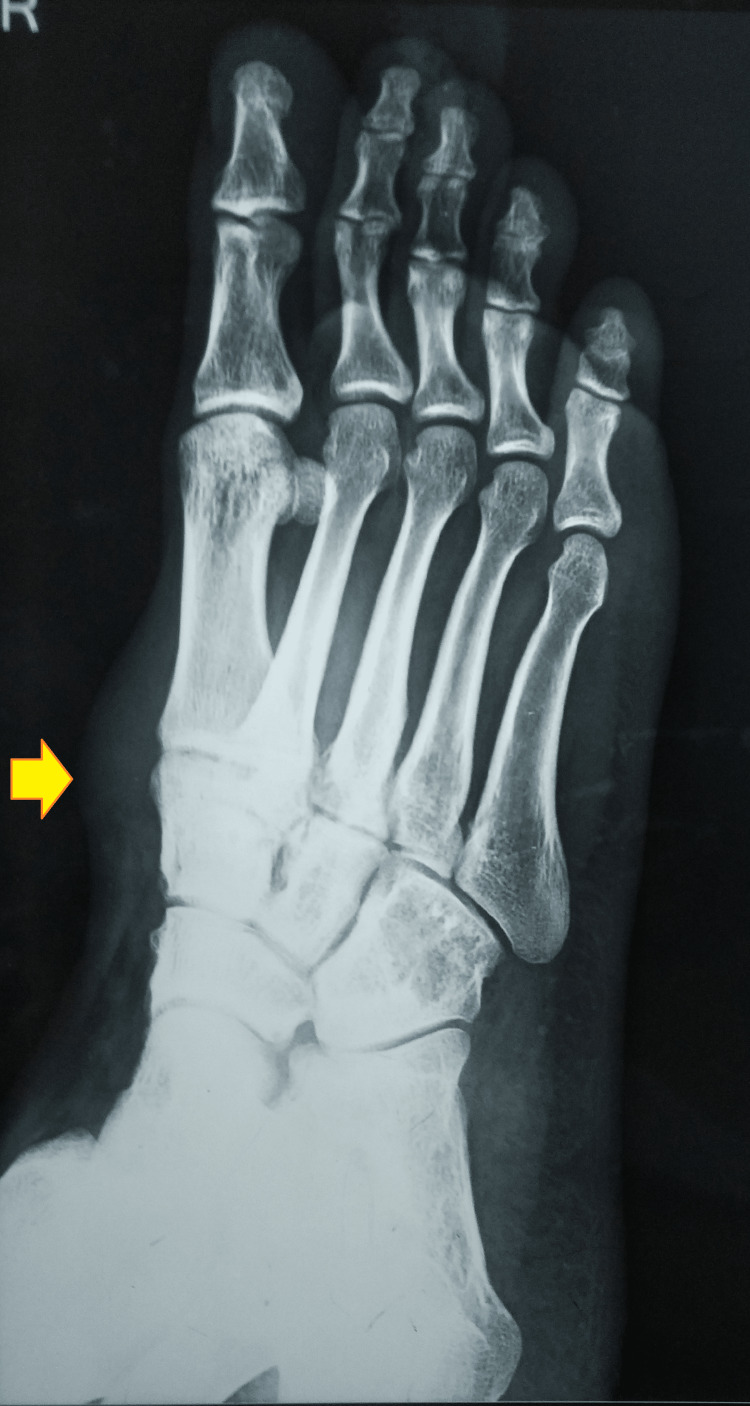
Oblique radiographs of the right foot showing the soft tissue swelling

The anteroposterior and oblique radiographs of the right foot were remarkable for soft tissue swelling with an osteolytic lesion and cortical thinning of the cuboid and fifth metatarsal base (Figures 3, 4).

An open biopsy and wound debridement with drainage of about 12 mL of thick, yellowish-colored pus were done. The biopsied samples were sent for histopathological and microbiological tests. The smear of aspirated pus for acid-fast bacilli on Ziehl-Neelsen staining was negative, but cartridge-based nucleic acid amplification tests detected *Mycobacterium tuberculosis* (low) with no resistance to rifampicin. The histopathology of the pus samples was suggestive of tuberculosis with epitheloid granulomas, caseating necrosis with occasional Langhans giant cells, and lymphocytic infiltrates. The line-probe assay and culture of the pus were negative. Advanced radiometric investigations like magnetic resonance imaging of the right foot were not done due to financial constraints and the patient's refusal.

As the diagnosis was established as primary tubercular osteomyelitis of the cuboid and fifth metatarsal without pulmonary involvement, he was put on anti-tubercular chemotherapy (Table 1).

**Table 1:** Anti-tubercular chemotherapy national guidelines

Phase	Drug	Dose	Duration	Route of administration
Intensive phase	Rifampicin	10 mg/kg	56 days	Per oral
	Pyrazinamide	25 mg/kg	56 days	Per oral
	Ethambutol	15 mg/kg	56 days	Per oral
	Isoniazid	5 mg/kg	56 days	Per oral
Continuation phase	Rifampicin	10 mg/kg	10 months	Per oral
	Ethambutol	15 mg/kg	10 months	Per oral
	Isoniazid	5 mg/kg	10 months	Per oral

His treatment also included a tablet of pyridoxine (1 mg/kg/day), dietary advice for a high protein intake, counseling for treatment adherence, and regular follow-up in the infectious disease and orthopedics outpatient departments. He responded well to the treatment for the first three months, with a reduction in pain and no obvious swelling. At his request, he was referred to a different district, and his last follow-up was not feasible. However, his outcome on the national tuberculosis portal showed treatment completion as his outcome.

## Discussion

Tuberculosis is a significant source of mortality and morbidity in endemic countries [1]. Often, pulmonary involvement is seen, which spreads to other body parts through lymphatic and hematogenous modes [8]. Osteoarticular tuberculosis is mainly reported in the spine (50-70%) (thoracic 50% > cervical 25% > lumbar 25%), pelvis (12%), hip and femur (10%), knee and tibia (10%), ribs (7%), ankle, foot, or shoulder (2%), elbow or wrist (2%), and multiple sites (3%) [5]. Tuberculosis affecting the small bones of the foot is very rare and is usually seen in less than 10% of cases of osteoarticular tuberculosis, which is about 0.1-0.3% of all extrapulmonary cases of tuberculosis [5]. Moreover, tuberculosis of the foot is commonly reported in the hindfoot and could present in four different forms, which are periarticular osseo-granuloma, central osseo-granuloma, primary hematogenous synovitis, and tenosynovitis and bursitis [5]. Further, tubercular osteomyelitis of the metatarsal has an incidence of less than 0.5%, with a proclivity to the first and fifth metatarsals [8].

Diagnosis of tuberculosis of the small bone is an onerous task [10]. It is due to issues like a late presentation, the paucibacillary nature of the disease, non-specific radiological features, the absence of specific serological markers, overlapping clinical features with other musculoskeletal disorders, and a lack of awareness among primary care clinicians [1,10]. Lack of access to advanced radiometric investigations like magnetic resonance imaging, computed tomography, etc. in the developing world results in delays in management [1].

Case reports of the involvement of the cuboid and metatarsals are available in the literature [1,5,8]; however, there is a paucity of data on the concomitant involvement of the cuboid and fifth metatarsal in the absence of pulmonary seeding, especially in an immunocompetent case.

Therefore, this case would serve as an important addition to the existing literature and would help clinicians in handling these cases. Further, large-scale data from heavy centers related to similar clinical presentations would help in modifying the current diagnostic and management algorithms.

## Conclusions

A non-diabetic 34-year-old Indian male is presented who was advised to undergo a battery of serological investigations along with radiographs to establish the diagnosis. He was managed with anti-tubercular treatment. It requires greater suspicion to diagnose these cases, as there are limited data available even in endemic countries. Besides, in the absence of a history of trauma and tuberculosis, it was a taxing diagnosis.
